# Inhibiting the MNK-eIF4E-β-catenin axis increases the responsiveness of aggressive breast cancer cells to chemotherapy

**DOI:** 10.18632/oncotarget.13772

**Published:** 2016-12-01

**Authors:** Zhiqiang Li, Yang Sun, Miao Qu, Hongxing Wan, Fang Cai, Peng Zhang

**Affiliations:** ^1^ Department of Oncology, Sanya People’s Hospital, Sanya 572000, China; ^2^ Special Care Unit, Sanya People’s Hospital, Sanya 572000, China; ^3^ Department of Pharmacy, Sanya People’s Hospital, Sanya 572000, China; ^4^ Department of Oncology, Tongji Hospital, Tongji Medical College, Huazhong University of Science and Technology, Wuhan 430030, China

**Keywords:** breast cancer, chemoresistance, MNK, eIF4E, β-catenin

## Abstract

Advanced breast cancer (eg. stage IV) is resistant to chemotherapy. In this work, we identified potentially druggable targets that are critically involved in chemoresistance. We showed that eIF4E is highly phosphorylated at serine 209 in breast cancer patients in response to chemotherapy, which significantly correlated with poorer clinical responses and outcomes. Depletion of eIF4E enhanced the anti-proliferative and pro-apoptotic effects of chemotherapeutic drugs in breast cancer cells. Chemotherapy activated the Wnt/β-catenin signaling in an eIF4E-dependent manner. However, MNK inhibitors prevented chemotherapeutic drug-induced eIF4E phosphorylation and β-catenin activation, which enhanced the breast cancer cell response to chemotherapy *in vitro* and *in vivo*. These findings indicate MNK-eIF4E-β-catenin is an activator of the breast cancer cell response to chemotherapy and highlights the therapeutic value of inhibiting MNK to overcome chemoresistance in breast cancer.

## INTRODUCTION

Breast cancer represents a major health problem and is one of leading causes of cancer related mortality in women [1]. Although standard treatments, including surgery, radiotherapy, chemotherapy, and anti-estrogen therapy significantly improve clinical responses and outcomes, advanced-stage breast cancer remains a major therapeutic challenge due to resistance to chemotherapy [2].

Eukaryotic translation initiation factor 4E (eIF4E) is essential for cap-dependent mRNA translation and represents a key regulatory node in the control of protein expression [3, 4]. eIF4E overexpression/activation correlates with poor clinical outcome in human cancers as it promotes the translation of carcinogenesis associated mRNAs [5, 6]. MAPK-interacting kinase (MNK) can directly regulate eIF4E phosphorylation in response to a variety of signals affecting tumor cell growth [7, 8]. Notably, MNK kinases are required for eIF4E phosphorylation at Ser209 in tumor cells, but not normal cells [9]. In addition, eIF4E knockdown reportedly decreases the breast cancer cell proliferation, and inhibition of MNK kinase activity by cercosporamide or CGP57380 effectively targets lung, prostate and breast cancer cells [7, 10–12]. However, the role of eIF4E phosphorylation in breast cancer resistance to chemotherapy is unknown.

In this study, we examined the phosphorylation level of eIF4E in breast cancer patients (stage IV) before and after chemotherapy, and assessed the role of eIF4E phosphorylation in the development of chemoresistance. Our work demonstrates that eIF4E phosphorylation is critically involved in chemoresistance and highlights the therapeutic potential of inhibiting MNK kinases to overcome resistance to chemotherapy in breast cancer patients.

## RESULTS

### eIF4E phosphorylation is a common feature in breast cancer patients’ response to chemotherapy and correlates significantly with poor clinical outcome

**Figure 1:**
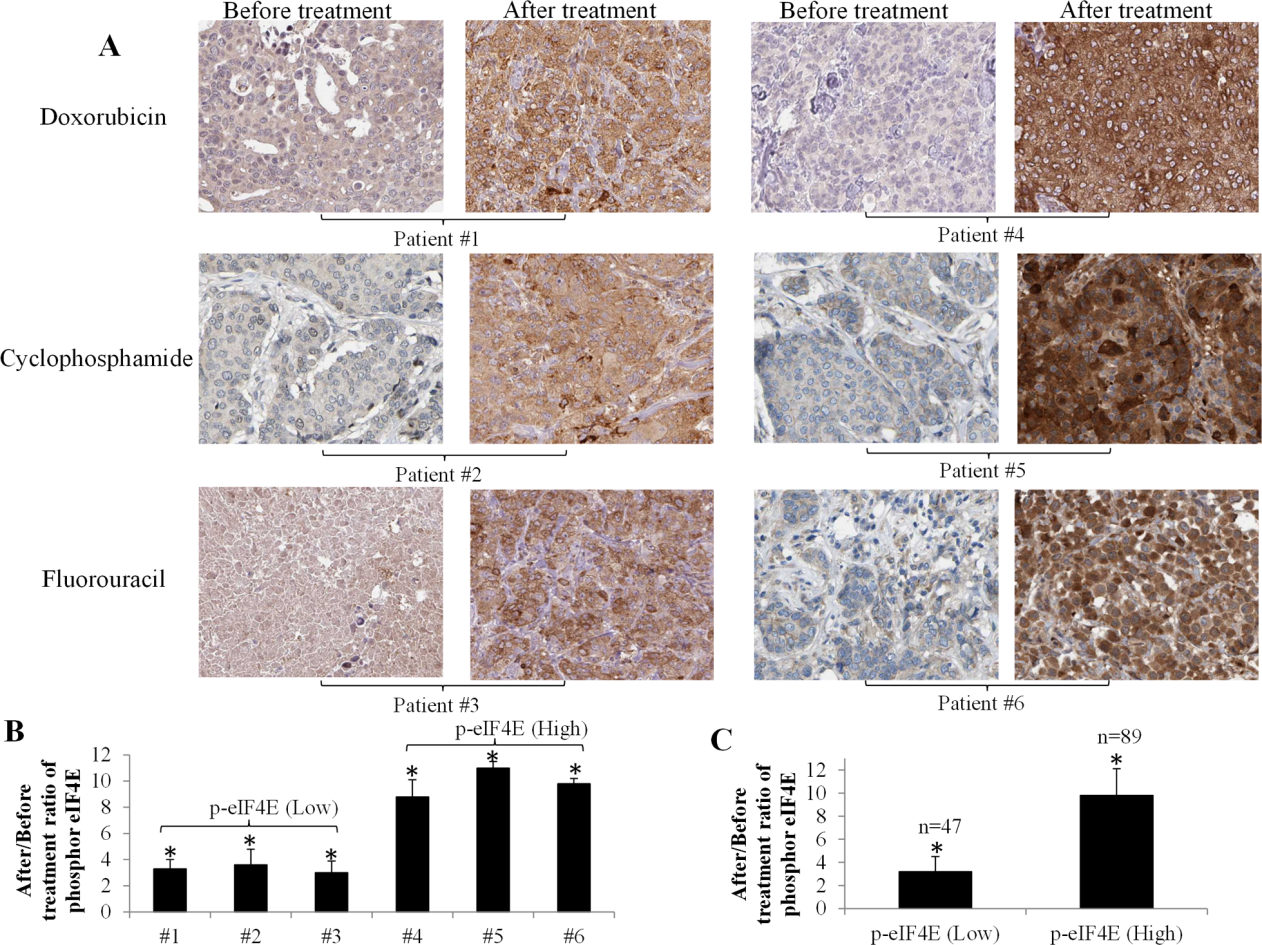
Significant increase of eIF4E phosphorylation in breast cancer patients after chemotherapy (**A**) Representative immunohistochemistry analysis shows eIF4E phosphorylation levels in breast cancer tissues from patients with stage IV breast cancer before and after chemotherapy. The patients received a minimum of 1 month of chemotherapy prior to biopsy for analysis of p-eIF4E levels. Original magnification 200X. (**B**) Quantification of immunohistochemical staining using Image J. Results shown are the ratios of values after/before treatment. Patients with the increased level of p-eIF4E by 2–4 fold are defined as p-eIF4E (Low) and patients with the increased level of p-eIF4E by 9–11 fold are defined as p-eIF4E (High). (**C**) Average of p-eIF4E levels in p-eIF4E(Low) and p-eIF4E (High) groups. Number of patients with p-eIF4E (Low) and p-eIF4E (High) are 47 and 89, respectively. *p < 0.05, compared to untreated controls or p-eIF4E (Low).

We first performed immunohistochemistry analysis on breast cancer tissues obtained from 200 patients diagnosed with stage IV breast cancer before and after chemotherapy. Supplementary Table S2 summarizes the baseline patient characteristics. Consistent with the previous report [11], we observed that the p-eIF4E levels were low (patient #1, #2 #3 and #4) or minimal (patient #5 and #6) in breast cancer patients before stating chemotherapy (Figure 1A). However, we found that phosphorylation levels of eIF4E at Ser209 were significantly increased in all patient samples received chemotherapy, such as doxorubicin, cyclophosphamide or fluorouracil (Figure 1A). This evidence demonstrates that eIF4E phosphorylation is a common feature in breast cancer patients’ response to chemotherapy. Quantification of tissue immunohistochemistry staining using Image J software indicates that phosphorylated (Ser209) eIF4E levels were increased by three to eleven fold in breast cancer patients after chemotherapy compared to the counterparts before chemotherapy (Figure 1B).

**Figure 2:**
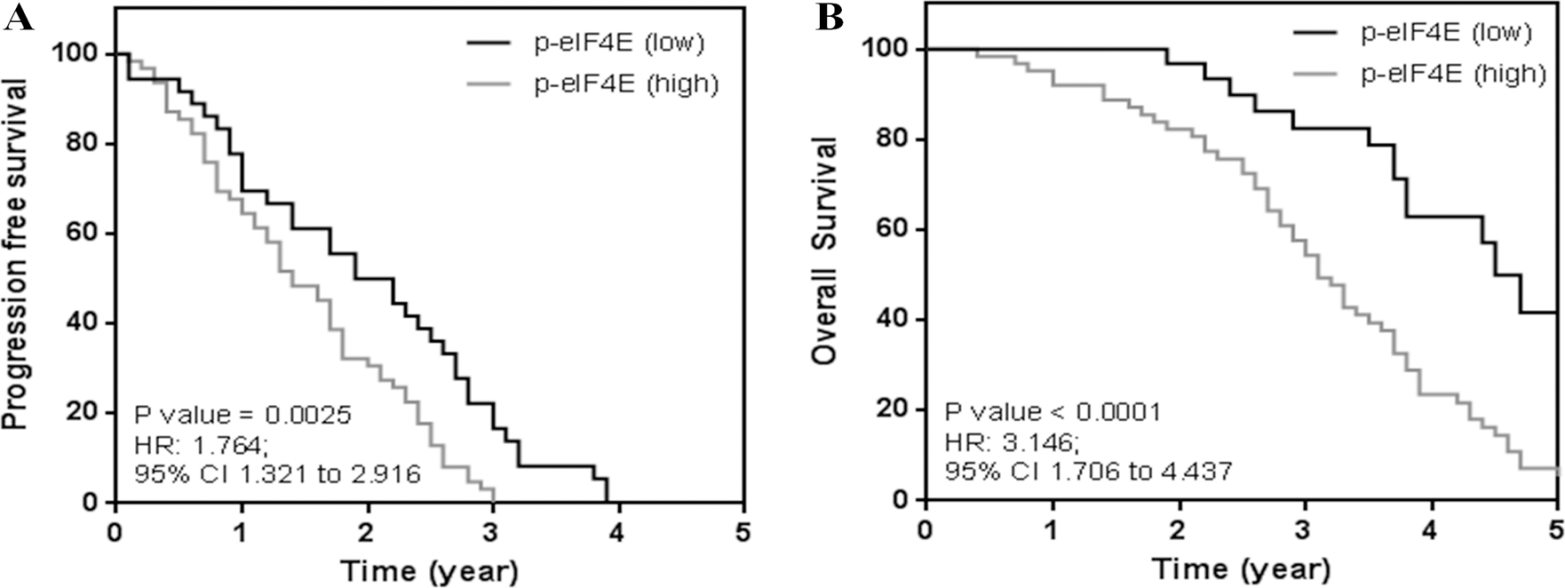
Kaplan meier analysis of breast cancer patient groups (**A**) Progression free survival showing patients with low levels of p-eIF4E have better clinical outcome. (**B**) Patients with low levels were observed to have better survival outcomes. Median overall survival for patient cohort (p-eIF4E (low)) was 4.7 years as compared to 3.1 years for the other study group.

We next classified the patients into two categories: the patients with the increased level of p-eIF4E by 2–4 fold are p-eIF4E (Low) and the patients with the increased level of p-eIF4E by 9–11 fold are p-eIF4E (High). We found that 89 and 47 among 200 patients are p-eIF4E (High) and p-eIF4E (Low), respectively (Figure 1C). We tracked the progression free and overall survival of these two patient groups using the Kaplan Meier analysis (Figure 2). The progression free survival (PFS) analysis (Figure 2A) showed a significant difference in the study cohorts with p-eIF4E (Low) patients having better outcome (*P* value = 0.0025) and hazard ratio (HR) determined as 1.76 (95% CI 1.32 to 2.92). Median overall survival (OS) for this group was 4.7 years compared with 3.1 years for the p-eIF4E (high) group (Figure 2B). Using the log rank test, there is statistical significance in OS outcome for the two groups of patients (*P* value < 0.0001). The HR was determined to be 3.15 (95% CI 1.71 to 4.44). Our results clearly demonstrate the clinical value of patient stratification associated with the levels of p-eIF4E.

### eIF4E phosphorylation promotes proliferation and activates β-catenin signaling in breast cancer cells

**Figure 3:**
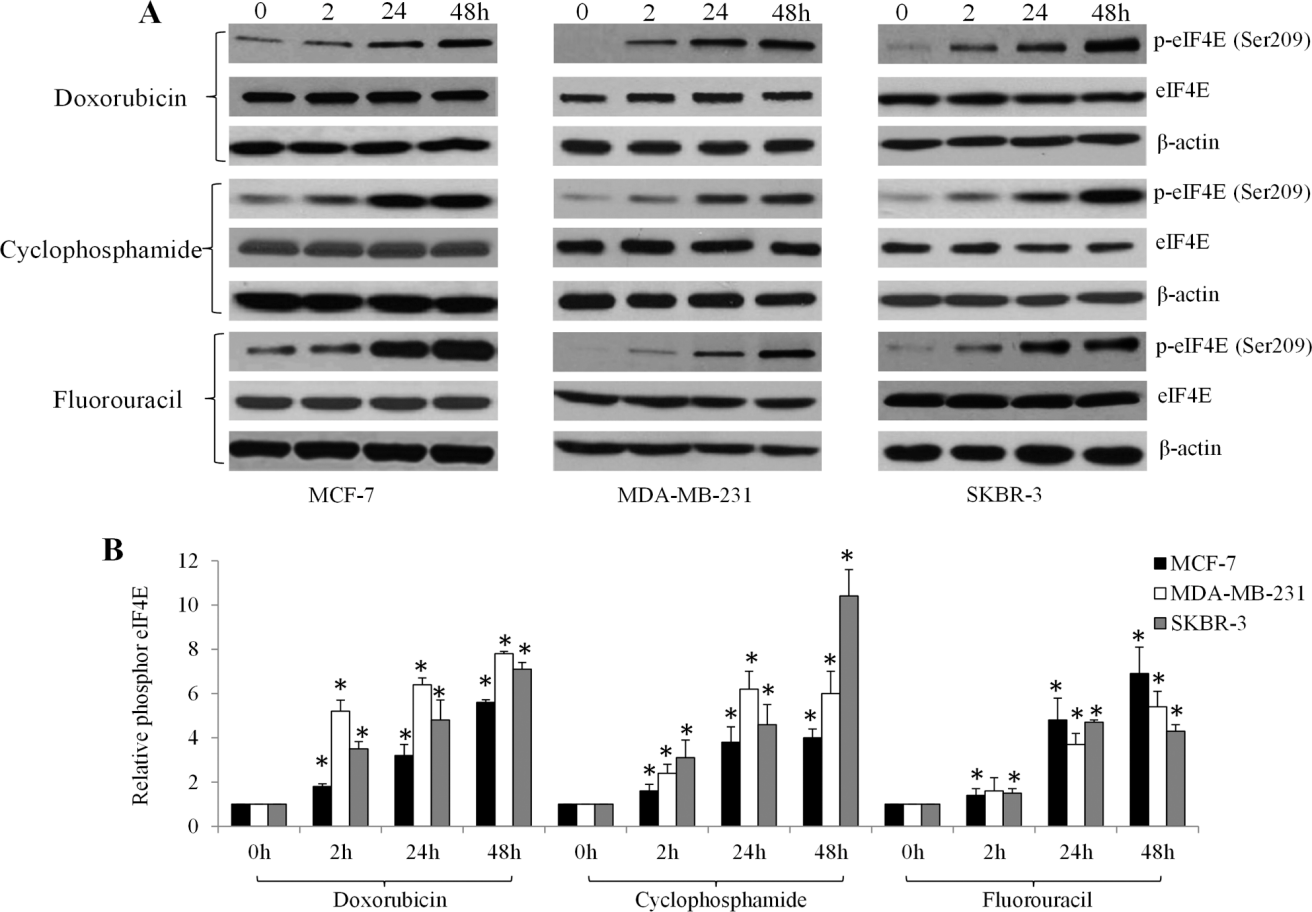
Chemotherapeutic drugs significantly increase eIF4E phosphorylation in breast cancer cells in a time-dependent manner Representative photos (**A**) and quantification (**B**) of immunoblot analysis show increased phosphorylated eIF4E in MCF-7, MDA-MB-231and SKBR-3 cells exposed to 10 μM doxorubicin, 20 μM cyclophosphamide and 10 μM fluorouracil.

**Figure 4:**
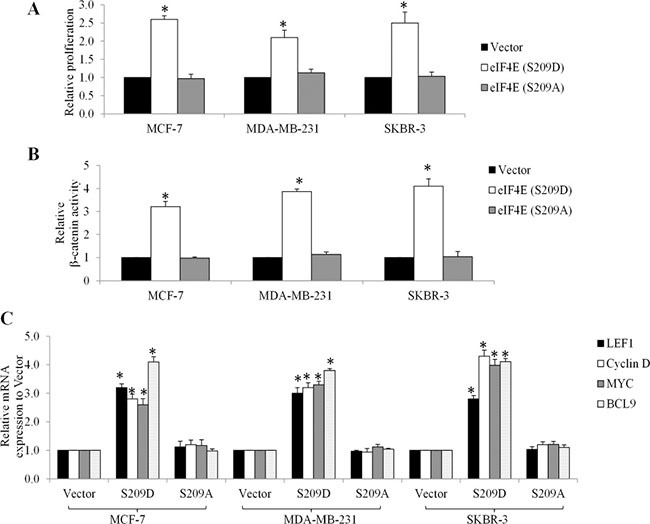
Overexpression of phosphor-mimetic eIF4E mutant (S209D) promotes growth and activates β-catenin signaling in breast cancer cells Overexpression of eIF4E (S209D) but not (S209A) promotes proliferation (**A**), increases β-catenin activity (**B**) and transcriptional mRNA levels of Wnt/ β-catenin-mediated genes (**C**) in MCF-7, MDA-MB-231and SKBR-3 cells. Results shown are expressed as fold relative to Vector. *p < 0.05, compared to Vector control.

Consistent with the patient data, western blot analysis showed that doxorubicin, cyclophosphamide and fluorouracil time-dependently increased eIF4E phosphorylation at Ser209 in several breast cancer cell lines, including MCF-7, MDA-MB-231, SKBR-3 cells (Figure 3). To understand the function of eIF4E phosphorylation in cancer cells, we used retroviral transduction to establish breast cancer cell lines overexpressing the phosphor-mimetic form of eIF4E (eIF4E S209D) or the non-phosphorylatable form (eIF4E S209A). We found that breast cancer cells with overexpression of eIF4E S209D (Ser to Asp), but not S209A (Ser to Ala) significantly grow faster than control cells (Figure 4A). In addition, we observed increased β-catenin activities in breast cancer cells overexpressing eIF4E S209D, but not S209A (Figure 4B). Consistent with the activation of β-catenin, the levels of Wnt/β-catenin-mediated transcription of target genes, including LEF1, Myc, Cyclin D and BCL9, were significantly increased (Figure 4C). These data suggest that activation of Wnt/β-catenin signaling is the consequence of eIF4E phosphorylation in breast cancer cells.

### Chemotherapy activates Wnt/β-catenin signaling in breast cancer cells in an eIF4E-dependent manner

**Figure 5:**
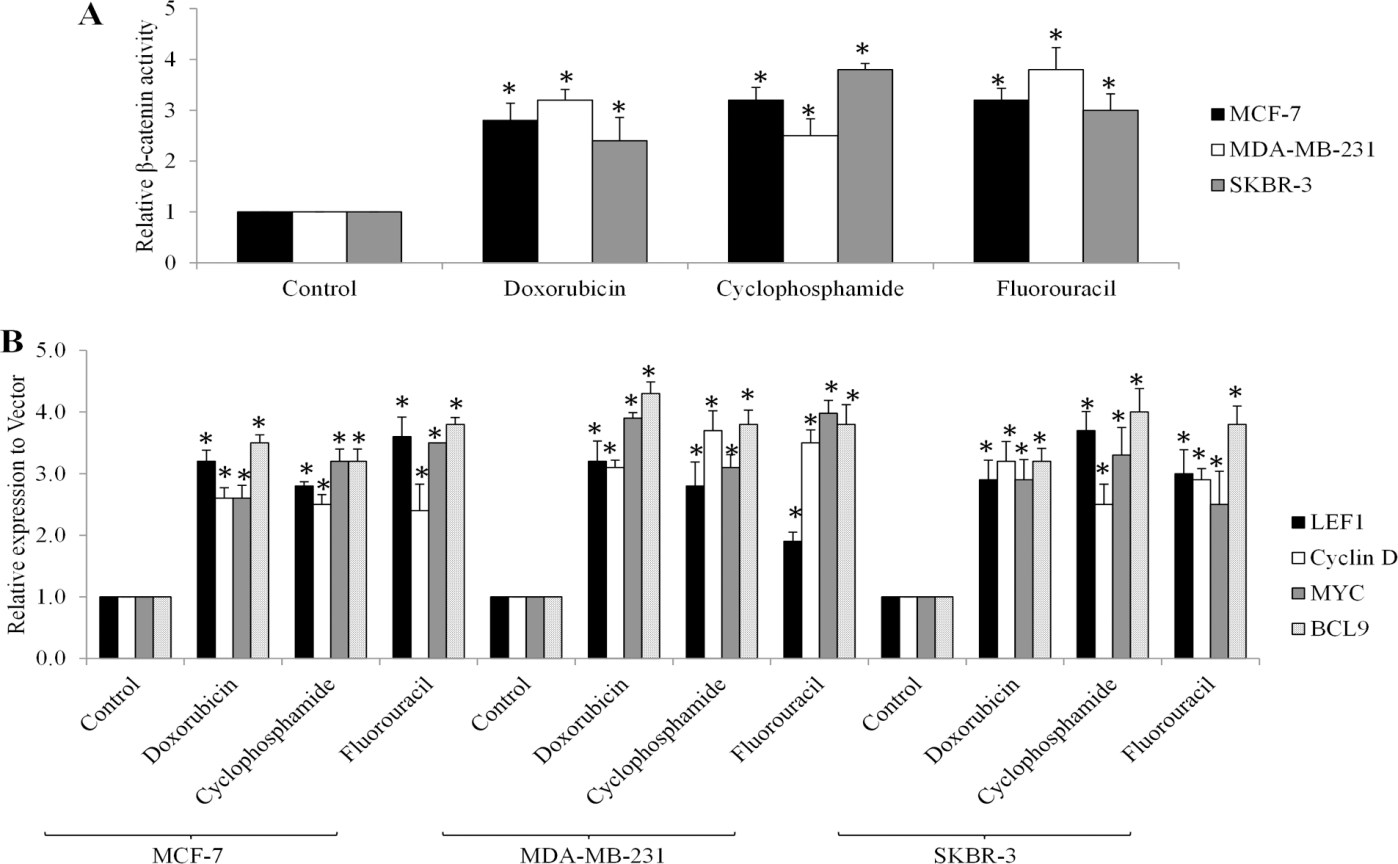
Chemotherapeutic drugs activate Wnt/β-catenin signaling in breast cancer cells Doxorubicin at 10 μM, cyclophosphamide at 20 μM and fluorouracil at 10 μM increases β-catenin activities (**A**) and mRNA levels of LEF1, Myc, Cyclin D and BCL-9 genes (**B**) in MCF-7, MDA-MB-231and SKBR-3 cells. *p < 0.05, compared to control.

Given that Wnt/β-catenin signalling activation is the consequence of eIF4E phosphorylation in breast cancer cells (Figure 4) and chemotherapy increases eIF4E phosphorylation (Figure 3), we next investigated whether chemotherapy induces the activation of Wnt/β-catenin signaling. We treated breast cancer cells with chemotherapeutic drugs and examined Wnt/β-catenin activities. We found that β-catenin activities and transcriptional mRNA levels of Wnt/β-catenin-mediated genes are significantly increased in breast cancer cells exposed to doxorubicin, cyclophosphamide or fluorouracil (Figure 5), demonstrating that chemotherapy activates Wnt/β-catenin signaling.

**Figure 6:**
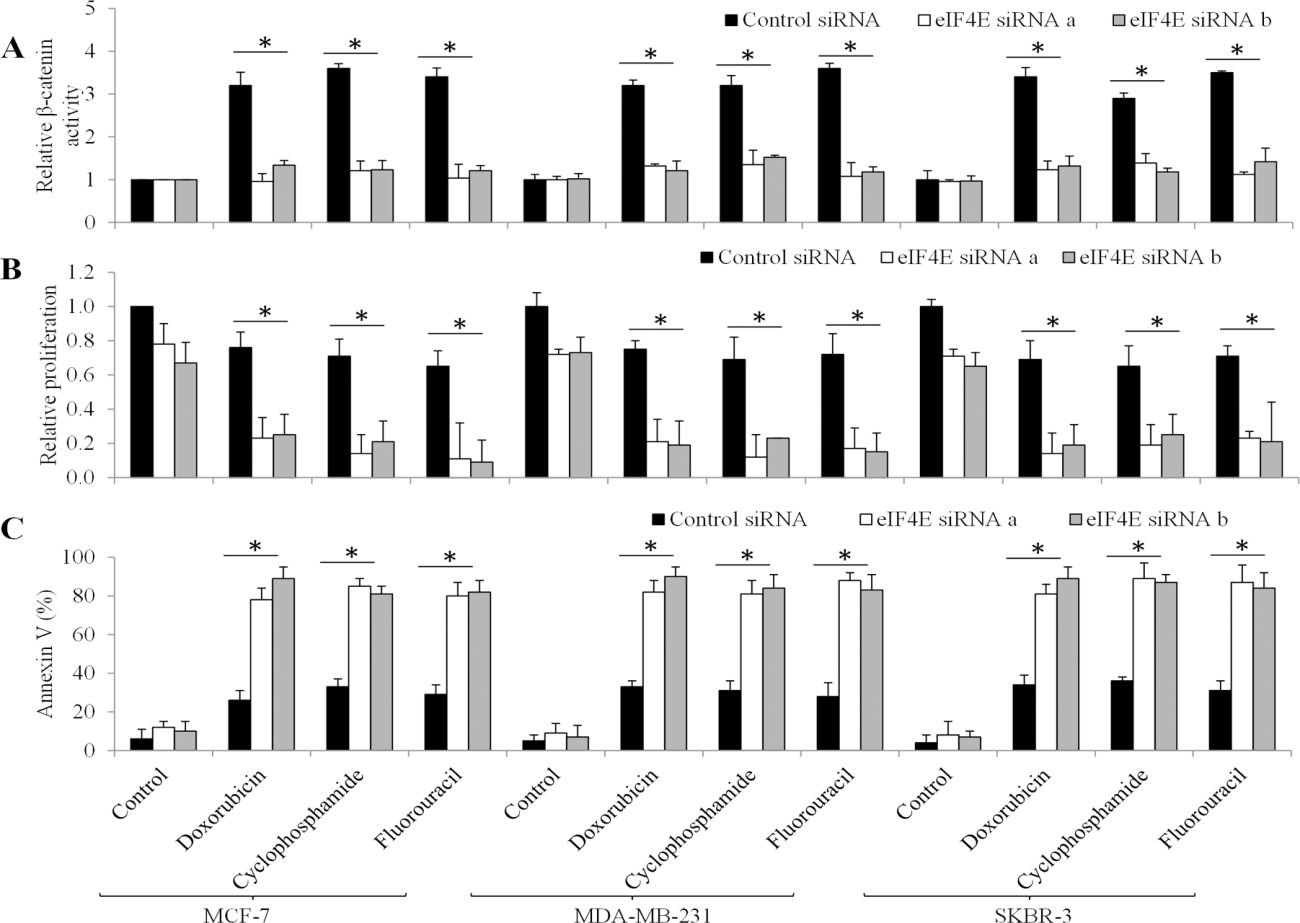
The effects of eIF4E knockdown in chemotherapeutic drugs-treated breast cancer cells (**A**) Doxorubicin, cyclophosphamide and fluorouracil fails to increase β-catenin activities in eIF4E-depleted breast cancer cells. Doxorubicin, cyclophosphamide and fluorouracil are more effective in inhibiting proliferation (**B**) and induce apoptosis (**C**) in eIF4E-depleted cells than control cells. Doxorubicin at 10 μM, cyclophosphamide at 20 μM and fluorouracil at 10 μM were used. *p < 0.05, compared to Control siRNA.

To further determine whether the activation of Wnt/β-catenin in breast cancer cells is eIF4E-dependent, we depleted eIF4E using two independent siRNAs in breast cancer cells (Supplementary Figure S1) and examined β-catenin activities in these cells exposed to chemotherapeutic drugs. We showed that doxorubicin, cyclophosphamide or fluorouracil failed to increase β-catenin activities in eIF4E-depleted breast cells (Figure 6A), demonstrating that chemotherapeutic drugs activate Wnt/β-catenin signaling in an eIF4E-dependent manner.

### Depletion of eIF4E augments the anti-proliferative and pro-apoptotic effects of chemotherapeutic drugs in breast cancer cells

To further understand the role of eIF4E in breast cancer cells in response to chemotherapeutic drugs, we analyzed the proliferation and apoptosis in eIF4E-depleted cells upon chemotherapeutic drugs treatment. The concentrations of drugs we used inhibited ~20% of proliferation and induced ~30% of apoptosis in control siRNA cells. However, chemotherapeutic drugs at the same concentration inhibited ~90% of proliferation and induced 85% of apoptosis in eIF4E-depleted breast cancer cells (Figure 6B and 6C).

### MNK inhibition suppresses β-catenin activities and enhances breast cancer cell response to chemotherapeutic drugs

**Figure 7:**
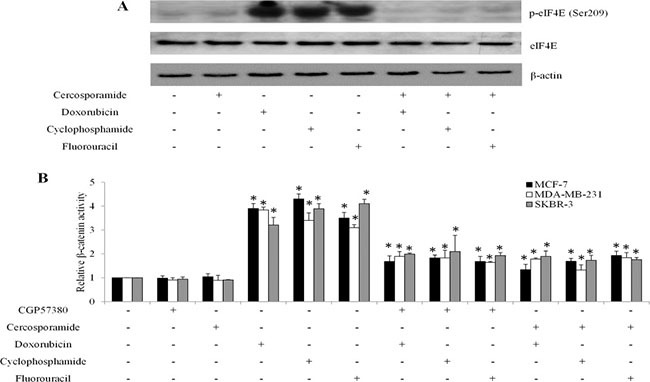
MNK inhibition abolishes chemotherapeutic drugs-mediated β-catenin activation in breast cancer cells (**A**) Representative western blot analysis show the levels of eIF4E phosphorylation in MCF-7 cells exposed to MNK inhibitors, chemotherapeutic drugs or both. (**B**) CGP57380 or cercosporamide inhibits β-catenin activation induced by chemotherapeutic drug in breast cancer cells . *p < 0.05, compared to control.

**Figure 8:**
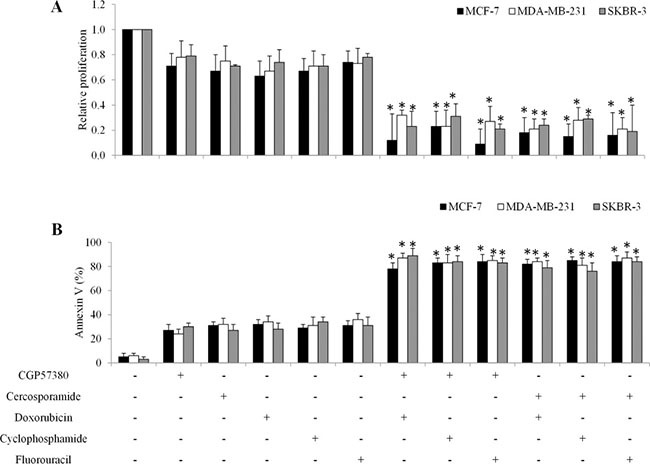
MNK kinase inhibitors sensitize breast cancer cells to doxorubicin in *in vitro* Combination of MNK inhibitors with chemotherapeutic drugs significantly inhibits more proliferation (**A**) and induces more apoptosis (**B**) than single drug alone in breast cancer cells. *p < 0.05, compared to single arm.

The MNK1/2 kinases are required for the phosphorylation of eIF4E at S209 *in vivo* [9]. To investigate whether the contribution of the MNK kinases to eIF4E phosphorylation was essential for β-catenin activity in breast cancer cells in response to chemotherapeutic drugs treatment, we exposed breast cancer cells to chemotherapeutic drugs or MNK kinase inhibitors (eg. CGP57380 and cercosporamide [7, 13]) alone, or combination. We found that CGP57380 decreased eIF4E phosphorylation induced by chemotherapeutic drugs in breast cancer cells (Figure 7A). In addition, CGP57380 or cercosporamide prevented the chemo drugs-induced eIF4E-mediated β-catenin increase in breast cancer cells (Figure 7B). We further found that CGP57380 or cercosporamide significantly augmented the anti-proliferative and pro-apoptotic effects of chemotherapeutic drugs (Figure 8). Collectively, our data indicate that MNK inhibition enhances the inhibitory effects of chemotherapeutic drugs in breast cancer cells, most likely by enhancing eIF4E phosphorylation and β-catenin activation.

### MNK inhibition enhances breast cancer cell response to chemotherapy *in vivo*

**Figure 9:**
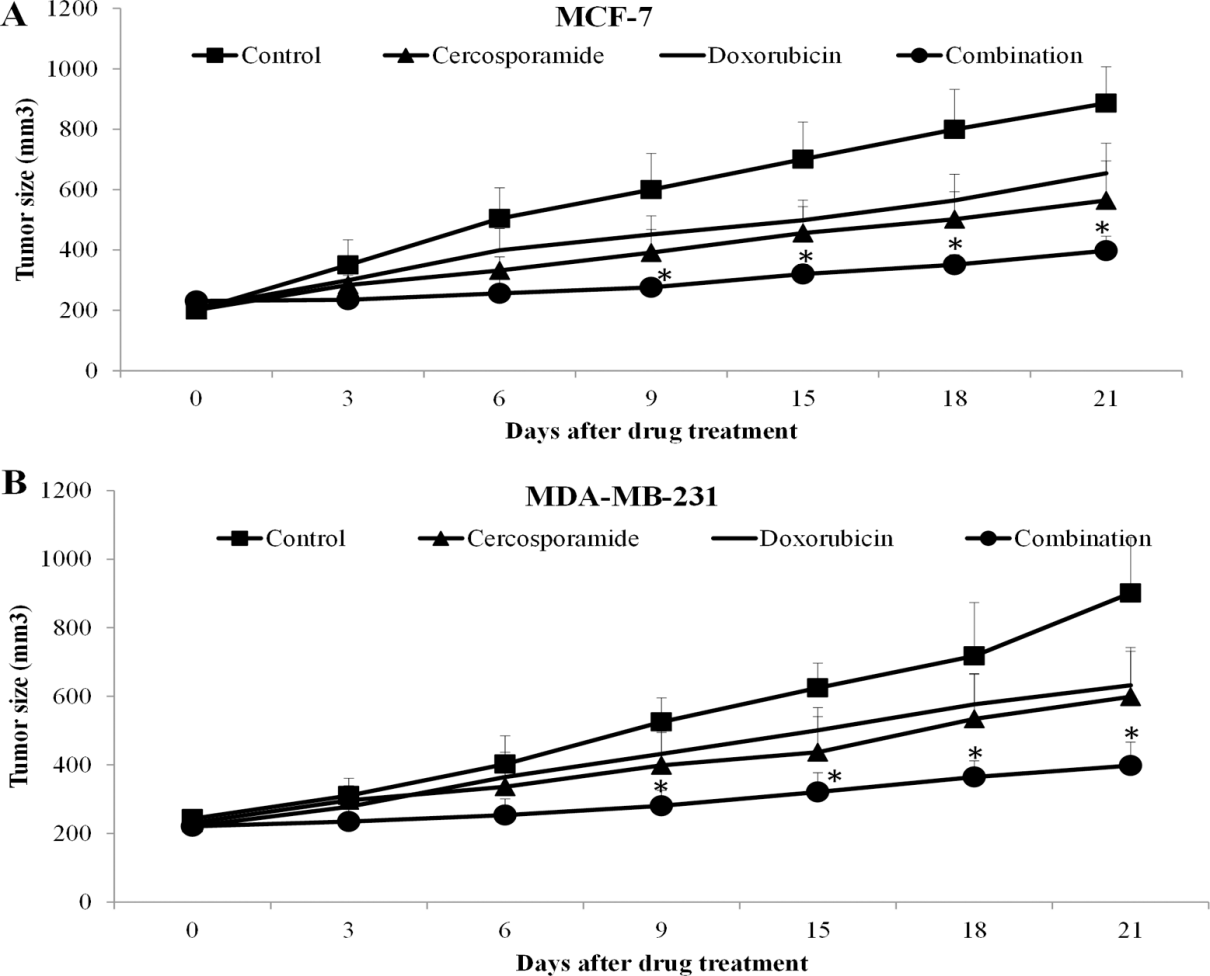
MNK kinase inhibitors sensitize breast cancer cells to doxorubicin *in vivo* Cercosporamide and doxorubicin slightly inhibits MCF-7 (**A**) and MDA-MB-231 (**B**) tumor growth as a single agent. Combination of both drugs more effectively inhibits tumor growth. There are ten mice in each group. *p < 0.05, compared to single arm.

Our findings suggested that MNK inhibitors sensitize breast cancer cell response to chemotherapy *in vitro*. To further explore this possibility in *in vivo*, we tested the combinatorial effects of doxorubicin and cercosporamide in two-independent breast cancer xenograft mouse models. Mice were tolerable to 40 mg/kg doxorubicin and 40 mg/kg cercosporamide as symptoms of toxicity were not observed (data not shown). Cercosporamide alone delayed MCF7 and MDA-MB-231 tumor growth (Figure 9). When combined with doxorubicin, the significantly higher efficacy in tumor growth arrest was observed (Figure 9).

## DISCUSSION

Further understanding of the molecular mechanism underlying chemoresistance is essential to the development of targeted therapy to overcome resistance in breast cancer. In our study, we showed that activation of the MNK-eIF4E-β-catenin axis is critically involved in the breast cancer cell response to chemotherapy. We also highlight the therapeutic value of inhibiting MNK kinases for overcoming chemoresistance in breast cancer.

We observed that eIF4E phosphorylation at S209 is a consistent feature in advanced breast cancer patients and in all tested breast cancer cell lines, including an ERα-positive line and HER2-, ERα- and PR-negative lines, which affects the response to chemotherapy (Figures 1 and 3). Importantly, eIF4E phosphorylation levels significantly correlate with poor clinical outcome (Figure 2). This finding confirms and extends the observations of McClusky et al. and Flowers et al., who reported that overexpression of eIF4E is associated with poor outcome and a higher rate of relapse in breast cancer [14, 15]. In particular, for the patient samples and cell lines we examined, all samples exhibited minimal p-eIF4E levels (Figures 1 and 3) prior to chemotherapy. This suggests eIF4E phosphorylation mainly plays a role in breast cancer cells’ response to chemotherapy rather than in disease development and progression.

A significant finding of this work is that chemotherapy-induced eIF4E phosphorylation leads to Wnt/β-catenin signaling activation. Both overexpression of eIF4E S209D and chemotherapeutic drugs activate Wnt/β-catenin by increasing β-catenin activity and Wnt/β-catenin-mediated transcription in breast cancer cells (Figures 4 and 5). Depletion of eIF4E abolishes the chemotherapeutic drugs-induced Wnt/β-catenin activation (Figure 6), which shows that Wnt/β-catenin activation functions as a consequence of eIF4E phosphorylation. eIF4E overexpression and phosphorylation reportedly regulates β-catenin in leukemia stem cells to maintain stem cell functions [16]. Overexpression of eIF4E S209D facilitates breast cancer cell growth and depletion of eIF4E augments the anti-proliferative and pro-apoptotic effects of chemotherapeutic drugs (Figures 4A and 6). Previous studies demonstrated that Mcl-1 and cyclin D are downstream targets of MNK-eIF4E [17, 18]. Mcl-1 and cyclin D are tumor-promoting genes that play essential roles in tumor progression, metastasis and resistance [17, 18]. Our work adds β-catenin to the list of eIF4E-targeted tumor promoting genes.

Importantly, the eIF4E-β-catenin axis is regulated by MNK kinases, as CGP57380 and cercosporamide prevent chemotherapy-induced eIF4E phosphorylation and β-catenin activation (Figure 7). In addition, CGP57380 and cercosporamide significantly augment the inhibitory effects of chemotherapeutic drugs in inhibiting proliferation and inducing apoptosis of breast cancer cells *in vitro* and *in vivo* (Figures 8 and 9). This suggests MNK inhibition effectively overcomes chemoresistance. Because MNK kinases are required for eIF4E phosphorylation at S209 in tumors, but not normal cells [9], MNK inhibitors may provide a selective therapeutic approach to treating breast cancer. However, a potential limitation of the current study is the lack of a clinical correlation for MNK inhibitors and will have to be addressed in future clinical interventional studies.

In sum, our findings emphasize the essential roles of MNK-dependent eIF4E-β-catenin activation in the breast cancer cell response to chemotherapy. Consequently, inhibiting MNK-eIF4E-β-catenin signaling using MNK inhibitors represents a potentially effective strategy for sensitizing breast cancer cells to chemotherapy.

## MATERIALS AND METHODS

### Patient, tissue specimens and immunohistochemistry

Two hundred female patients with breast cancer hospitalized at Sanya People’s Hospital and Tongji Hospital between year 2005–2008 were included in the study Supplementary Table S2. All patients included were diagnosed with stage IV breast cancer and received no treatment at the time of diagnosis. Written informed consent was obtained from all patients under institutional review board approved protocols. This study was approved by the ethics committee of Sanya People’s Hospital and Tongji Hospital. Representative tissue sections were deparaffinised with xylene and hydrated through ethanol into water. Antigen retrieval was conducted using 10% citrate acid buffer. Sections were stained with p-eIF4E (Cell Signalling Technologies, US) and then secondary antibody conjugated with HRP. Sections were finally counterstained with haematoxylin.

### Cell culture, generation of cell lines and drugs

Human breast cancer cell lines, MCF-7, MDA-MB-231 and SKBR-3, were purchased from American Type Culture Collection (ATCC) and cultured in Minimal Essential Media (MEM) containing 10% fetal bovine serum (Hyclone, UK) and 2 mM L-glutamine (Invitrogen, US). Cell lines stably overexpressing eIF4E mutant forms were established by retroviral transduction using MSCV-internal ribosome entry site (IRES) constructs as previously described [19]. Doxorubicin (D1515, Sigma, US), cyclophosphamide (C7397, Sigma) and dasatinib (F6627, Sigma) were dissolved in H_2_O and DMSO, respectively. CGP57380 and cercosporamide were purchased from CalBiochem and dissolved in DMSO.

### Cell proliferation and apoptosis assays

Cell were treated with single drugor combinations for 72 hours. Cell proliferation activity was evaluated by using the CellTiter 96 AQueous One Solution Cell Proliferation assay kit (Promega, US) according to manufacturer’s instructions. Assessment of apoptosis was performed by staining cells with Annexin V-FITC and 7-AAD (BD Pharmingen, US) followed by flow cytometry on a Beckman Coulter FC500.

### Denaturing sodium dodecyl sulfate–polyacrylamide gel electrophoresis (SDS–PAGE) and Western blot (WB) analyses

Cells were homogenized in RIPA lysis buffer (Invitrogen, US) containing 1× protease inhibitor cocktail (Roche, US). Equal amount of total protein extracts were separated by SDS-PAGE and then processed for Western blot analysis by using antibodies recognizing eIF4E, phosphor-eIF4E (S209) and β-actin (Cell Signalling Technology, US).

### TOPflash report assay

Cells were transfected with 5 μg of M50 Super 8x TOPFlash plasmid (a kind gift from Dr. Randall Moon [20] ) or 5 μg of β-gal by using Dharmafect Transfection Reagent. At 24 h after transfection, cells were harvested by using the Luciferase Report Assay System (Promega, US) or the β-gal Enzyme Assay System (Promega) according to manufacturer’s instructions. β-catenin activity was calculated by the TOPflash after normalization of β-gal.

### Real time PCR

The total RNA was isolated with TRIzol Reagent (Ambion, US). The first-strand cDNA was synthesized by using iScript cDNA Synthesis Kit (Bio-rad, CA) and used to perform PCR using a SsoFast EvaGreen Supermix and CFX96 RT PCR system (Bio-rad, CA). The primers used for PCR was listed in Supplementary Table S1.

### siRNA transfection

eIF4E knockdown were performed in cells using two independent siRNAs targeting different regions of eIF4E. One million cells were transfected with 100 nM scramble siRNA (siCtrl) or human eIF4E-specific siRNAs in the presence of Dharmafect Transfection Reagent. Cells were harvested for Western blot, report assays or cellular assays 24 h after transfection. The target sequences are the same as previously described [12].

### Breast xenograft in SCID mouse

All procedures were conducted according to the guidelines approved by the Institutional Animal Care and Use Committee. SCID mice were subcutaneously injected with 100 μl of five million cells suspended in PBS. When tumour volume reached ~200 mm^3^, the mice were treated with 40 mg/kg intraperitoneal cercosporamide, 40 mg/kg oral doxorubicin or combination once per day. The control group was treated with intraperitoneal 50%/50% DMSO/saline. Tumour volume was calculated using formula: (length) × (width)^2^/2.

### Statistical analyses

The data were obtained from at least three independent experiments and expressed at mean and standard deviation (SD). Statistical analyses were performed by unpaired Student’s *t* test for cell assays. To evaluate the clinical utility of measuring p-eIF4E levels, PFS and OS of the patients were compiled and analyzed using the Kaplan Meier Estimate. The HR comparing different patient groups was determined using the log rank test. *P*-value < 0.05 considered statistically significant. The statistical analysis for PFS and OS were calculated using the Prism Software (GraphPad, USA).

## SUPPLEMENTARY MATERIALS TABLES AND FIGURE


