# Supplementary material for: Inhibiting the MNK-eIF4E-β-catenin axis increases the responsiveness of aggressive breast cancer cells to chemotherapy
Source: Oncotarget. 2016 Dec 1;8(2):2906–15. doi: 10.18632/oncotarget.13772 (PMC5356851; doi:10.18632/oncotarget.13772)
Supplement: Supplementary file 1 [file oncotarget-08-2906-s001.pdf]

Inhibiting the MNK-eIF4E-β-catenin axis increases the responsiveness of aggressive breast cancer cells to chemotherapy

Supplementary Materials

Supplementary Table S1: Primer sequences

	Forward	Reverse
MYC	5'-AAT GAA AAG GCC CCC AAG GTA GTT ATC C-3'	5'-GTC GTT TCC GCA ACA AGT CCT CTT C-3'
Cyclin D	5'-CCG TCC ATG CGG AAG ATC-3'	5'-ATG GCC AGC GGG AAG AC-3'
BCL9	5'-AGA GAG AAG CAC AGC GCC TC-3'	5'-CTG CAG TCT GGT ATT CTG GGA AG-3'
LEF1	5'-AAC GAA CGA GAC TCT GGC ATG-3'	5'-CGG ACA TCT AAG GGC ATC ACA-3'
GAPDH	5'-AAC GGG AAG CTT GTC ATC AAT GGA AA-3'	5'-GCA TCA GCA GAG GGG GCA GAG-3'

Supplementary Table S2: Patient characteristics

		(N = 200)
**Median Age (years)**		58
**ECOG Performance Status**	0	98
	1/2	102
**Disease stage**	IV	200
**Histology**	Ductal	145
	Lobular	55
**Metastatic sites**	Bone only	19
	Visceral	107
	Others	74

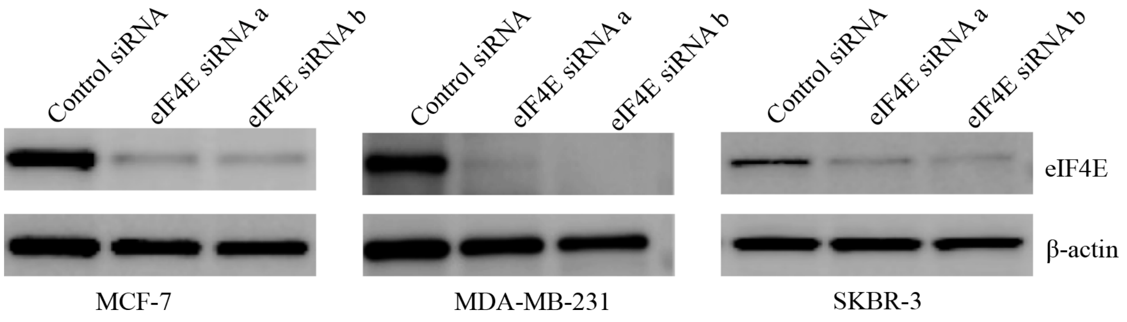

Supplementary Figure S1: Expression levels of eIF4E in the breast cancer cells transfected with eIF4E siRNA.
